# Supplementary material for: From sequence to enzyme mechanism using multi-label machine learning
Source: BMC Bioinformatics. 2014 May 19;15:150. doi: 10.1186/1471-2105-15-150 (PMC4229970; doi:10.1186/1471-2105-15-150)
Supplement: Additional file 2 — Java code of ml2db. Additional file ml2db_code.tar.gz contains the Java source code to run the multi-label machine learning experiments and save the results to database. The code’s Javadoc is included. [file 1471-2105-15-150-S2.zip › additional file 2/ml2db/ml2db/doc/main/ml2db/mulan/mulanarff/MulanDataSetGenerator.html]

MulanDataSetGenerator


JavaScript is disabled on your browser.


- Overview
- Package
- Class
- Use
- Tree
- Deprecated
- Index
- Help

- Prev Class
- Next Class

- Frames
- No Frames

- All Classes

- Summary:
- Nested |
- Field |
- Constr |
- Method

- Detail:
- Field |
- Constr |
- Method


main.ml2db.mulan.mulanarff

## Class MulanDataSetGenerator

- java.lang.Object
- - main.ml2db.weka.DataSetGenerator
  - - main.ml2db.mulan.mulanarff.MulanDataSetGenerator

- ---

    

  ```
  public class MulanDataSetGenerator
  extends DataSetGenerator
  ```

  Class

  Version:
  :   8 Sep 2010

  Author:
  :   Luna De Ferrari ldf(at)st-andrews.ac.uk

- - ### Constructor Summary

    Constructors

    Constructor and Description

    `MulanDataSetGenerator(MulanDataSetManager mulanDataSetManager,
    InstAttValueMap instanceAttributeMap,
    InstAttValueMap instanceClassMap)`
  - ### Method Summary

    Methods

    | Modifier and Type | Method and Description |
    | --- | --- |
    | `MulanAttributeFactory` | `getAttributesFactory()` |
    | `int` | `getNumberOfAttributes()` Get the number of distinct feature attributes (all attribute values - class values) |
    | `int` | `getNumberOfInstanceAttributeCouples()` Get the number of distinct instance-attribute couples of values by subtracting the number of instance-class\_attribute values from the total number. |

    - ### Methods inherited from class main.ml2db.weka.DataSetGenerator

      `fillInstances, getAttributes, getClassName, getInstanceAttributeMap, getInstanceClassMap, getInstances, getInstancesFactory, getInstancesNames, getManager, getNumberOfClassValues, getNumberOfInstanceClassCouples, getNumberOfInstances, getNumberOfInstancesFromDataMaps, getNumberOfInstancesWithClass, getTempFile, makeInstances, setInstancesNames`
    - ### Methods inherited from class java.lang.Object

      `equals, getClass, hashCode, notify, notifyAll, toString, wait, wait, wait`

- - ### Constructor Detail


    - #### MulanDataSetGenerator

      ```
      public MulanDataSetGenerator(MulanDataSetManager mulanDataSetManager,
                           InstAttValueMap instanceAttributeMap,
                           InstAttValueMap instanceClassMap)
      ```
  - ### Method Detail


    - #### getAttributesFactory

      ```
      public MulanAttributeFactory getAttributesFactory()
      ```

      **Overrides:**
      :   `getAttributesFactory` in class `DataSetGenerator`


    - #### getNumberOfAttributes

      ```
      public int getNumberOfAttributes()
      ```

      Get the number of distinct feature attributes (all attribute values -
      class values)

      **Overrides:**
      :   `getNumberOfAttributes` in class `DataSetGenerator`

      Returns:


    - #### getNumberOfInstanceAttributeCouples

      ```
      public int getNumberOfInstanceAttributeCouples()
      ```

      Get the number of distinct instance-attribute couples of values by
      subtracting the number of instance-class\_attribute values from the total
      number.

      **Overrides:**
      :   `getNumberOfInstanceAttributeCouples` in class `DataSetGenerator`


- Overview
- Package
- Class
- Use
- Tree
- Deprecated
- Index
- Help

- Prev Class
- Next Class

- Frames
- No Frames

- All Classes

- Summary:
- Nested |
- Field |
- Constr |
- Method

- Detail:
- Field |
- Constr |
- Method
